# Unveiling the mechanism of metabolites derived from gut microbiota in the treatment of acute kidney injury via network pharmacology and bioinformatics

**DOI:** 10.1186/s40643-026-01122-4

**Published:** 2026-09-02

**Authors:** Weiguo Yao, Jinlin Huo, Kun liu, Pengyu Tao

**Affiliations:** 1 Department of Nephrology, Jinshan District Central Hospital Affiliated to Shanghai University of Medicine and Health Sciences, Shanghai, China; 2 Clinical Medical Research Center, The First Affiliated Hospital of Shantou University Medical College, Shantou, China

**Keywords:** Gut microbiota, Metabolites, AKI, Bioinformatics, Network pharmacology

## Abstract

**Background:**

Acute kidney injury (AKI) affects a considerable proportion of patients that represents a major challenge for clinical treatment. Gut microbiota metabolites have been reported to attenuate acute kidney injury (AKI), yet their underlying mechanisms remain largely elusive. The present study aimed to explore the protective mechanisms of these metabolites against AKI using.

**Methods:**

The targets of metabolites and AKI were obtained from public databases. The PPI network is utilized to identify the core targets. GO and KEGG enrichment analysis were employed to predict significant pathway and biological functions. The Gut-Metabolites-Targets-Pathway network (G-M-T-P) network was constructed to screen the core metabolites. Furthermore, molecular docking was applied to evaluate the binding affinities between the metabolites and their candidate targets.

**Results:**

A total of 84 overlapping targets between gut microbiota metabolites and AKI were acquired. The beneficial effects of gut microbiota metabolites were associated with the regulation of inflammatory response and hypoxia. HIF-1 pathway was identified as the significant pathway mediating the effects of metabolites. STAT3, HIF1A, NFKB1, IL6 and AKT1 were identified as the core therapeutic targets of metabolites against AKI. The G-M-T-P network identified acetate, butyrate, propionate, and 3-indolepropionic acid as the core metabolites. The core metabolites exhibited strong affinity binding affinity for the core targets, suggesting their potential application in candidate drug development.

**Conclusion:**

The study highlights that the promising effects of gut microbiota metabolites in AKI treatment through modulating multi targets and pathways.

**Graphical abstract:**

**Figure:**
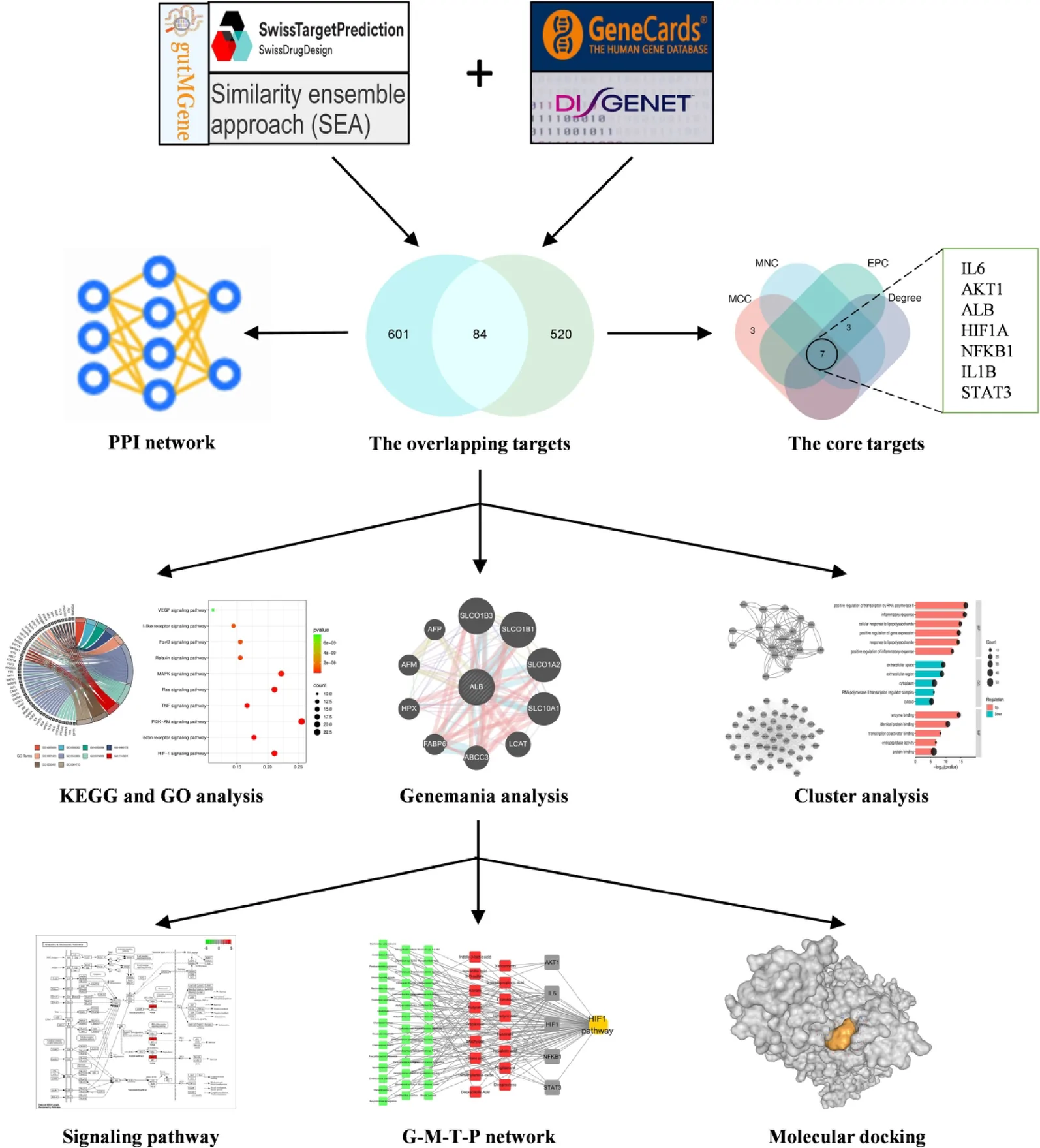

## Introduction

Acute kidney injury (AKI), one of the most serious global health challenges, is associated with high incidence and death rates (Livingston et al. 2023). The clinical manifestation of AKI is featured by the abrupt deterioration in renal function accompanied by the elevated level of serum creatinine (Scr) and decreased urine output (André et al. 2023). AKI triggers long-term complications. The survivors of AKI have a high likelihood of progressing to chronic kidney disease (CKD), and these CKD patients may even develop end-stage renal disease (ESRD) (Kuwabara et al. 2022; Miao et al. 2024). Current management remains limited to supportive care such as fluid balance adjustment or the kidney dialysis, which could not effectively reverse renal damage or halt the progression of disease (Dong et al. 2021). This progressive renal dysfunction severely impairs patients’ physical function, undermines their mental health, and substantially diminishes their overall quality of life(Dong et al. 2021). Therefore, there is an urgent need to develop new targeted interventions and the identify effective therapeutic targets that represent critical priorities for advancing AKI therapy.

Recent studies have demonstrated that the gut microbiota (GM)-kidney axis plays a pivotal role in the pathogenesis and progression of acute kidney injury (AKI) (He et al. 2025; Zhou et al. 2025), serving as a complex bidirectional communication network that links intestinal microbial homeostasis to renal function (Jin et al. 2026; Winner et al. 2025). Some risk factors like aging, antibiotic abuse or high-fat dietary intake often induce GM dysbiosis, which compromises the integrity of the intestinal barrier, enhances gut permeability, and permits the translocation of pathogen-associated molecular patterns (PAMPs) into the systemic circulation (Mali et al. 2025; Huai and Ye 2025). The lipopolysaccharide (LPS), one of the PAMPs, is a potent inflammatory inducer that triggers the activation of downstream pro-inflammatory signaling pathways (NF-κB and MAPK) via binding to Toll-like receptors (TLRs) (Liu et al. 2026), particularly TLR4 expressed on renal tubular epithelial cells (Li et al. 2022; Hu et al. 2024). These pro-inflammatory pathways act as key regulators that elicit robust renal inflammation, oxidative stress and tubular cell apoptosis, which are recognized as key hallmarks of AKI. Recently, short-chain fatty acids (SCFAs) were reported to exert protective effects by suppressing inflammatory related signaling pathways, promoting regulatory T cell differentiation and preserving renal tubular mitochondrial function via G-protein-coupled receptor activation and histone deacetylase (HDACs) inhibition (Li et al. 2022). Notably, a study demonstrated that SCFA administration attenuated ischemia-reperfusion-induced AKI in mice by restoring intestinal barrier function through upregulation of tight junction proteins and modulating systemic immunity via a reduction in pro-inflammatory monocytes and an increase in the abundance of healthy GM metabolites (Chou et al. 2022; Yang et al. 2024; Tian et al. 2024). Additionally, clinical evidence indicates that probiotic supplementation and fecal microbiota transplantation (FMT) can alleviate the severity of AKI and accelerate renal recovery by increasing the production of acetate and butyrate (Zhang et al. 2025). These GM metabolite-targeted interventions hold promise as potential preventive or adjunctive therapeutic strategies for AKI.

**Fig. 1:**
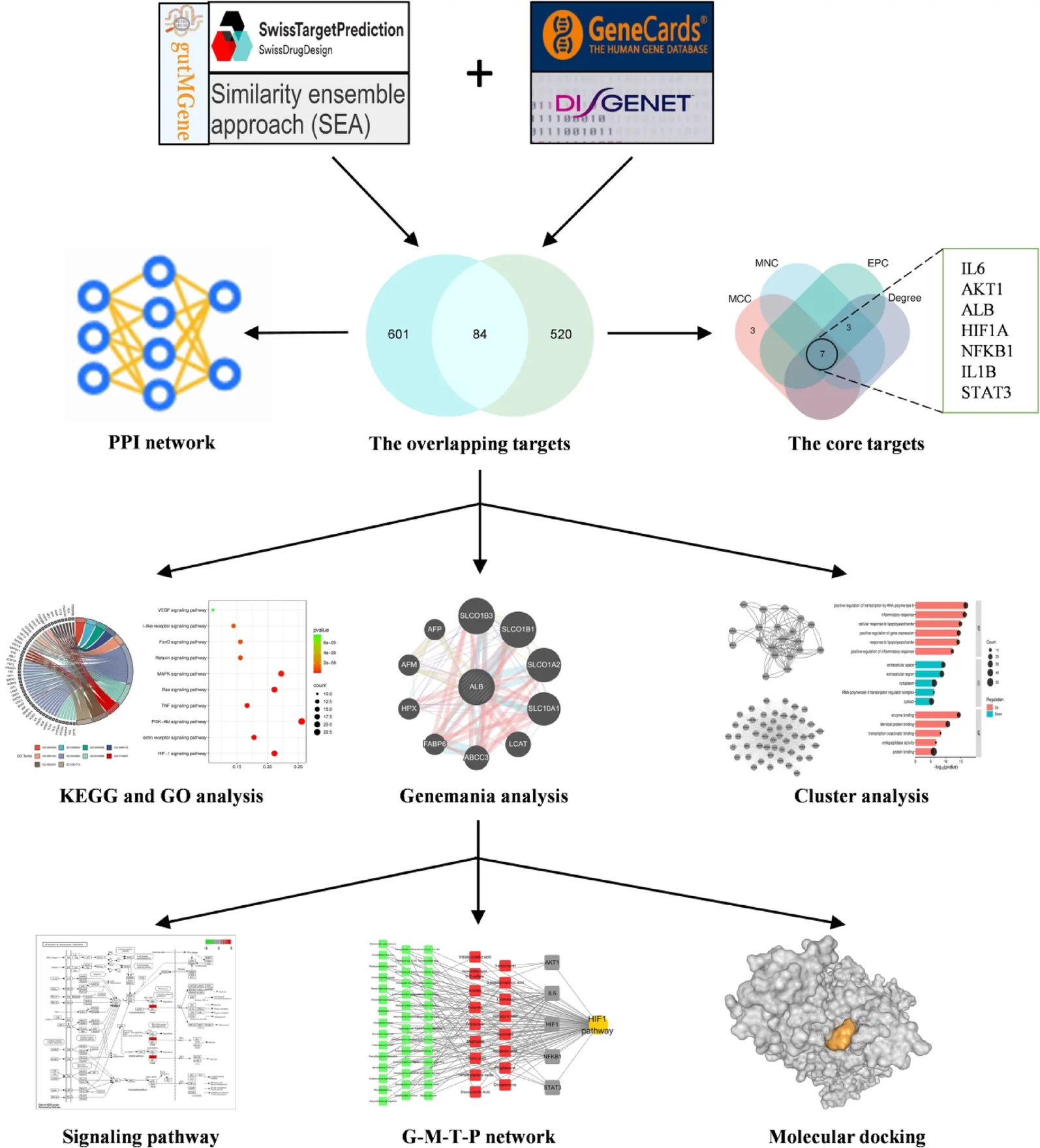
The flowchart of the study design. First, targets of gut microbiota metabolites were predicted using the SEA and STP. Second, AKI-related targets were retrieved from GeneCards and DisGeNET databases. The overlapping targets were identified by VENN diagram. KEGG and GO enrichment analysis were performed. PPI network used to screen core targets. The GeneMANIA functional association network was constructed to expand target dataset. Finally, the Gut Microbiota–Metabolites -Targets–Pathways(G-M-T-P) network was established to screen core metabolites

It is worthy of investigating the critical role of metabolites in the treatment of AKI. Network pharmacology is an emerging computer-based network analytical approach that involves the combination of multiple technologies such as bioinformatics enrichment analysis, protein–protein interaction (PPI) network analysis, and even topological analysis (Yao et al. 2025; Yao et al. 2025). The aim of this is to investigate the potential therapeutic mechanisms of GM metabolites on AKI through integrating network pharmacology, KEGG pathway prediction, GO enrichment analysis and molecular docking. We believed that this novel analytical approach enabled us to gain deep insights into the beneficial effects of GM metabolites from a new angle. The flowchart of this study was briefly described in Fig. 1.

## Methods and materials

### Data source and tool

The detailed information of data source and tool was available in Table 1.

**Table 1:** Database, software and analysis platform

NO	Database and analysis platform	Source	Version
1	gutMGene	http://bio-annotation.cn/gutmgene/	–
2	Swiss Target Prediction	http://www.swisstargetprediction.ch/	–
3	Pubchem	https://pubchem.ncbi.nlm.nih.gov/	–
4	bioinformatics	http://www.bioinformatics.com.cn/	–
5	STRING	https://string-db.org	–
6	Cytoscape software	https://cytoscape.org/	–
7	OMIM	https://omim.org/	–
8.	Genecards	https://www.genecards.org/	\
9	DisgeNet	https://www.disgenet.org/	–
10	DAVID Bioinformatics	https://david.ncifcrf.gov/tools.jsp	–
11	Similarity Ensemble Approach	https://sea.bkslab.org/	–

### The collection of gut microbiota metabolites targets

The metabolites derived from gut microbiota were obtained from gutMGene database, and their SMILE forms were download from PubChem. The Similarity Ensemble Approach (SEA) and Swiss Target Prediction (STP) were utilized to predict the targets of metabolites based on their SMILE forms. Subsequently, targets from SEA and STP were subjected to VENN diagram to acquire the overlapping targets, which are considered the targets collection of metabolites.

### The identification of potential targets of AKI

The targets of AKI were obtained from GeneCards and DisGeNET database by imputing keywords “AKI” and “Acute kidney injury” into the platform. The targets associated with AKI from the two database were merged after discarding the repeated targets, which used to further investigation.

### The Protein-Protein interaction (PPI) network

The overlapping targets were uploaded to the STRING platform to construct a protein-protein interaction (PPI) network. Subsequently, the PPI network was visualized using Cytoscape 3.6 software. The CytoHubba plugin was employed to identify crucial targets within this network leveraging multiple analytical algorithms, including the maximal clique centrality (MCC), maximum neighborhood component (MNC), and degree methods. The intersection targets were ultimately recognized as the core targets.

### GO function and KEGG enrichment analysis

The overlapping targets were submitted to DAVID platform for performing GO and KEGG analysis. GO function contains 3 parts: BP (Biological process), CC (Cellular Component) and MF (Molecular Function). KEGG enrichment analysis is used to identified potential pathways. We used “*P* value and FDR” to screen the most related GO terms or pathways that could better understanding the influence of metabolites on AKI. False discovery rate (FDR) threshold < 0.05, *P* < 0.05. MCODE plugin was used to analyze the functional cluster based on the following settings: Degree cut off: 2, Node score cutoff: 0.2, K-core: 2.

### Molecular docking verification

The structure files of core components and core targets were obtained from RCSB Protein Data Bank (RCSB PDB) and Pubchem database respectively. The target protein and ligand were separated by the PyMOL software. The pdbqt formats of target proteins, ligands and ingredients were prepared by AutoDock Tools. For the next step, the active pocket of the receptor was built with Grid plugin. Finally, the binding energy of the target protein and the active component was calculated by Vina.

## Results

### The targets of GM metabolites against AKI

**Fig. 2:**
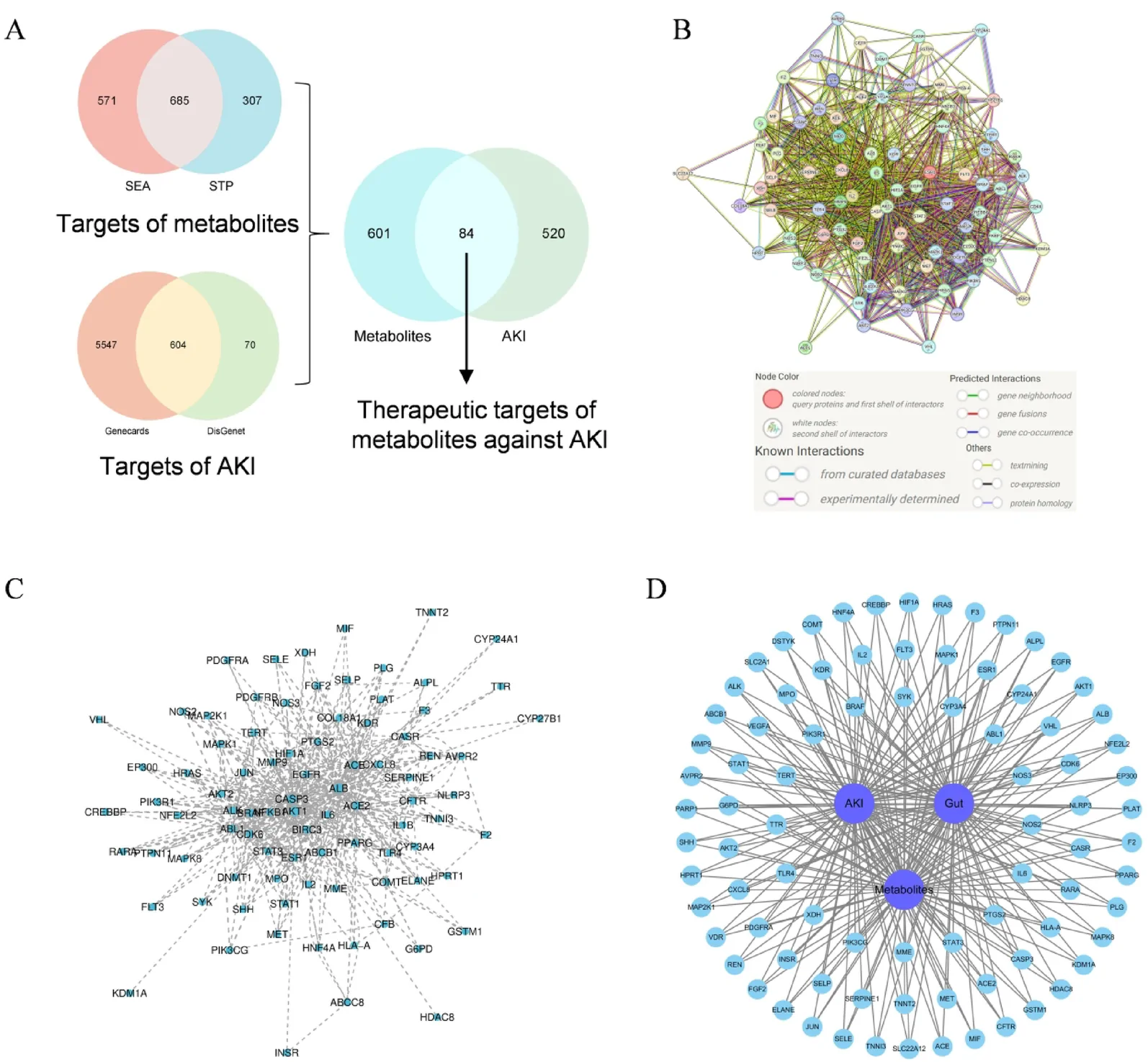
The screening of potential targets. **A** The VENN diagram displayed the overlapping targets between GM metabolites and AKI. **B** The PPI network analysis. **C** The visualization of PPI network analysis. **D** The Gut-Metabolites-AKI-Targets network

Targets associated GM metabolites’ prediction was performed via the SEA and STP databases, yielding 1256 and 992 candidate targets respectively (Fig. 2A). Subsequently, VENN diagram was conducted to generate 685 targets collection from the SEA and STP databases that considered as the key targets mediating the biological functions of gut microbiota metabolites (Fig. 2A). Next step, the same way was used to acquire the overlapping targets retrieved from the GeneCards and DisGeNet databases, yielding a total of 604 targets associated with AKI (Fig. 2A). Finally, a last round of intersection analysis was performed to identify a total of 84 overlapping targets that regarded as the hub targets of metabolites and AKI (Fig. 2A). To further elucidate the relationship among the hub targets, the PPI network was constructed with the STRING platform, which contained 84 nodes and 2680 edges (Fig. 2B and C). The lines with different colors indicated the targets might be associated the gene fusions, text mining, co-occurrence and co-expression. We also built a GM-metabolites-AKI network, demonstrating that these targets played a vital role in orchestrating the therapeutic actions of metabolites on AKI (Fig. 2D).

### Construction of PPI network for identifying core target

**Fig. 3:**
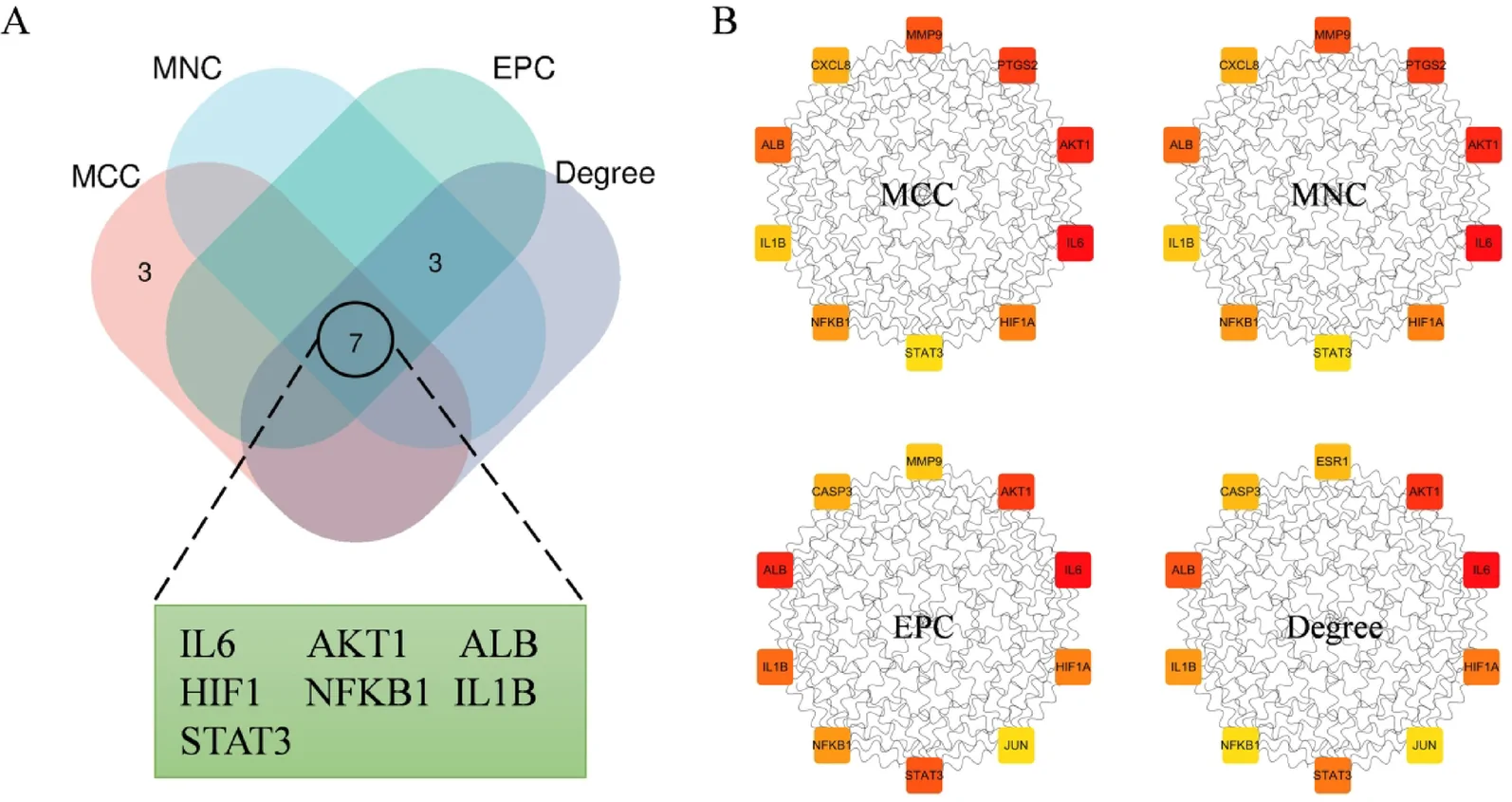
The identification of core targets. **A** The core targets acquired from 4 algorithms using VENN diagram. **B** Top 10 core targets acquired by four topological algorithms (MNC, MCC, EPC and Degree) in CytoHubba plugin

To further screen the core therapeutic targets, we employed multiple screening approaches using Cytohubba plugin with the Cytoscape software. MNC, MCC, EPC and Degree are four important screening approaches. Through employing the four important screening approaches, we successfully acquired the top 10 targets from each method (Fig. 3B). After intersecting with targets obtained via the four screening approaches, the seven overlapping targets (IL6, AKT1, ALB, HIF1A, NFKB1, IL1B, and STAT3) were identified as core targets (Fig. 3A), which implies their potential contributory functions in AKI treatment.

### GO and KEGG enrichment analysis of targets against AKI

**Fig. 4:**
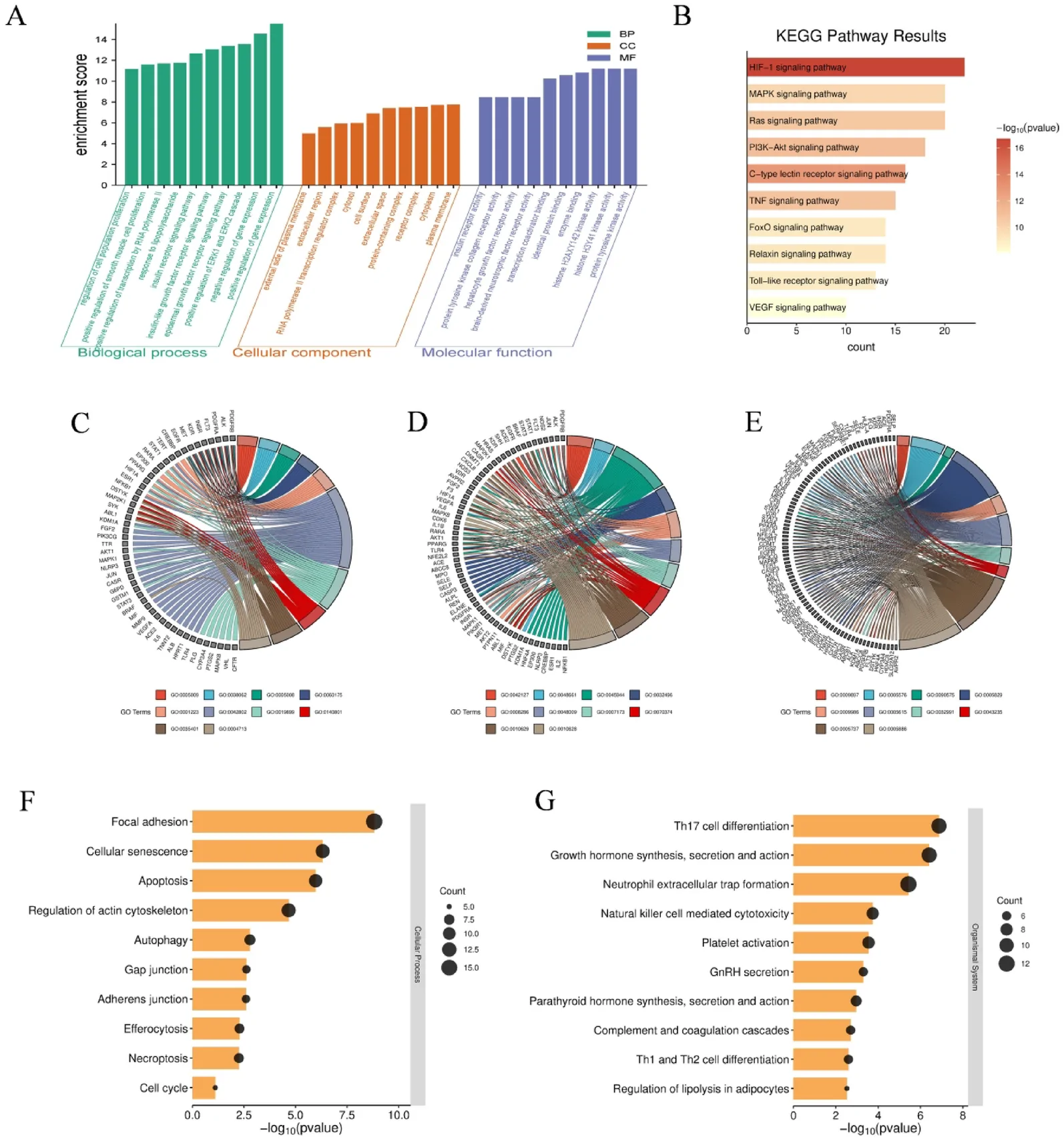
The GO and KEGG enrichment analysis. **A** The top 10 entries enriched in biological process (BP), cellular component (CC), and molecular function (MF). **B** The top 10 signaling pathways enriched in KEGG analysis. **C**, **D** and **E** The genes involved in the regulation of BP, CC and MF. **F** The top 10 entries in Cellular Process. **G** The top 10 entries in Organismal System

The GO enrichment analysis were categorized into biological process (BP), cellular component (CC), and molecular function (MF) (Fig. 4A). The top 10 BPs were primarily enriched in the regulation of cell proliferation, insulin/insulin-like growth factor receptor signaling pathways, and ERK1/ERK2 cascade regulation, which are central to renal tubular epithelial cell injury, repair, and inflammatory responses in AKI. For CC, significant enrichment was observed in the plasma membrane, cell surface, and transcription regulator complexes, reflecting subcellular changes in membrane integrity and transcriptional remodeling during AKI. In MF, genes were mainly enriched in protein tyrosine kinase activity, insulin receptor activity, and histone kinase activity, confirming the critical role of signal transduction and potential epigenetic regulation in AKI pathogenesis. The KEGG bubble plot also highlighted that the signaling pathways were related to VEGF, TNF, MAPK, PI3K-Akt and HIF-1(Fig. 4B). These pathways exerted multi functions including autophagy, apoptosis, cell differentiation, cell cytotoxicity and etc. (Fig. 4F and G). The chord diagram visually illustrates the multidirectional associations between targets and functional terms, indicating that a single target might simultaneously participate in multiple pathological processes such as inflammation activation, hypoxia and cellular repair (Fig. 4C-E). Collectively, these findings highlight that the targets and pathways may represent key regulatory mechanisms in AKI by modulating multiple biological processes, cellular components and molecular functions.

### GeneMANIA based network analysis of core targets of GM metabolites

**Fig. 5:**
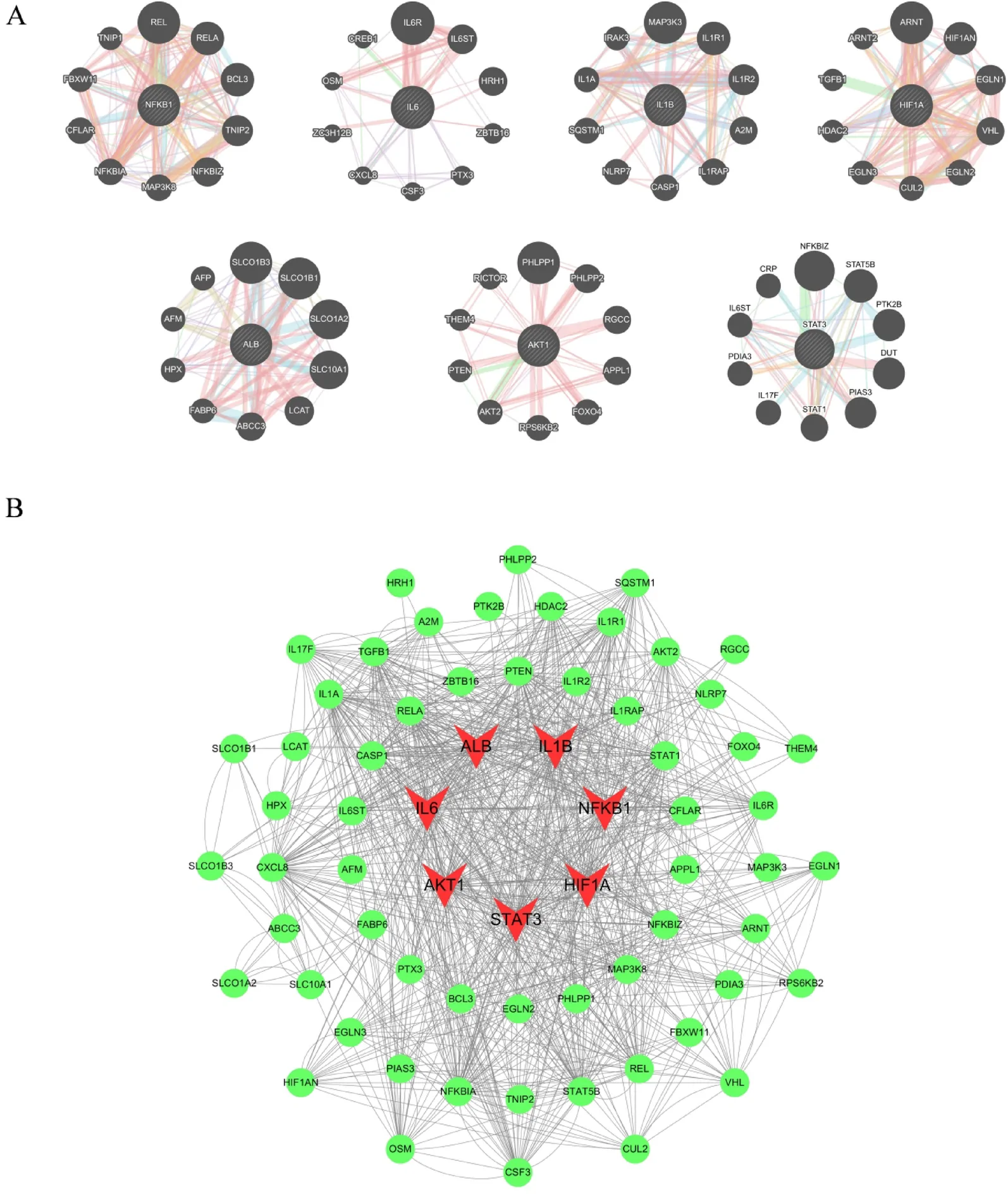
GeneMANIA based network (GMN) analysis. **A** The expanded target dataset of the 7 core targets with GMN analysis. **B** The network of 7 core targets and their expanded dataset

The GeneMANIA based network (GMN) analysis could uncover more vital information of targets relevant to the disease process by employing multiple evaluation parameters such as co-expression, genetic interaction, and physical interaction. According to our previously established GMN analysis method, we expanded the 7 core targets’ list by adding 10 additional genes to each target (Fig. 5A), which is termed the GMN expanded dataset (GMN-ED). The GMN-ED was visualized by uploading the data file into Cytoscape, which consisted of 66 nodes and 924 edges (Fig. 5B). The red node represented the core targets, while the green node represented their expanded targets. The GMN-ED was instrumental in providing solid evidence for the functional role of the identified targets in the subsequent enrichment analysis.

### The functional enrichment analysis of GMN-ED

**Fig. 6:**
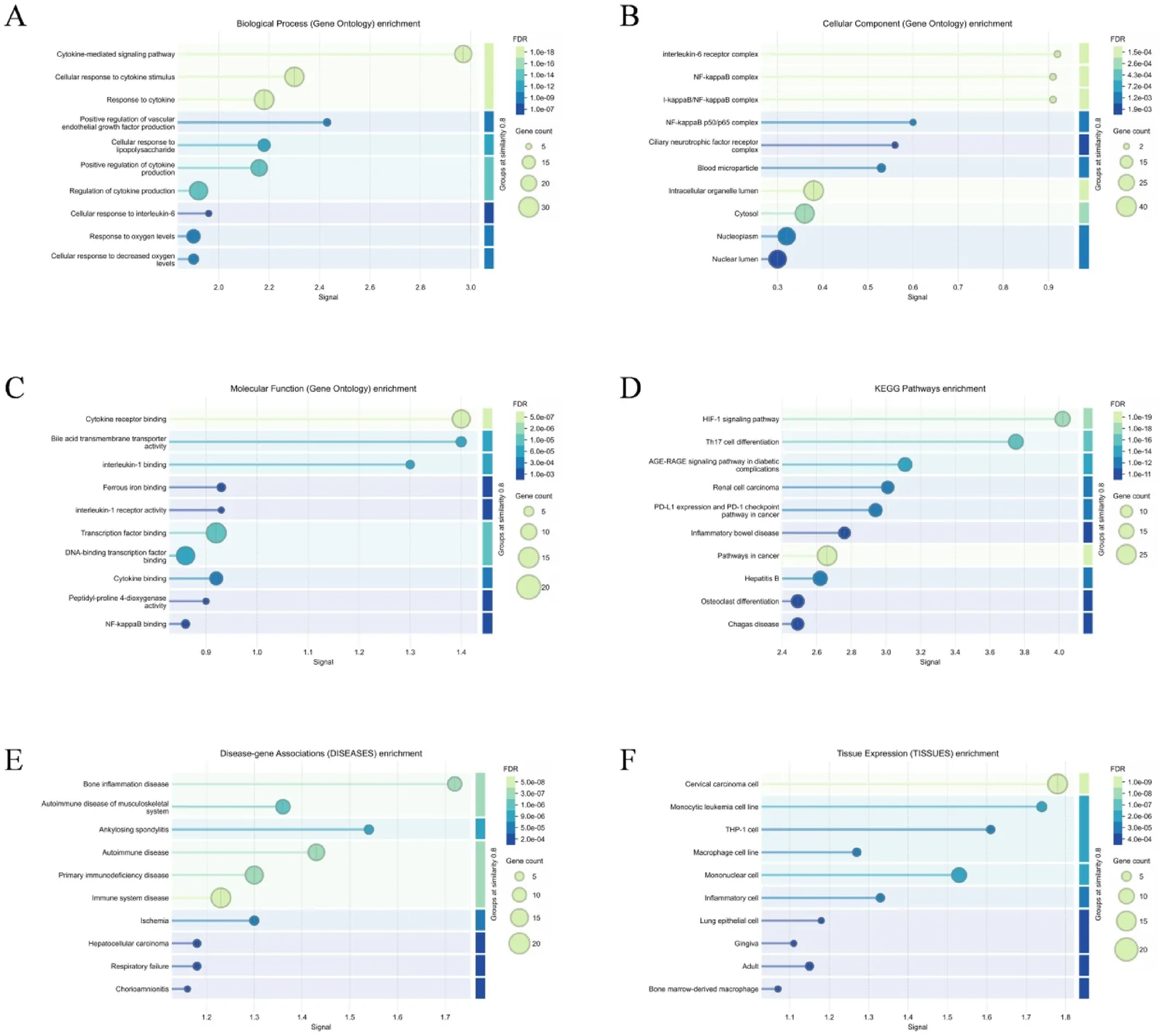
GO and KEGG enrichment analysis of targets identified in GMN. **A** The top 10 entries in BP. **B** The top 10 entries CC. **C** The top 10 entries MF. **D** The top 10 signaling pathways. **E** The genes involved in the regulation of diseases. **F** The genes enrichment in Tissue expression

**Fig. 7:**
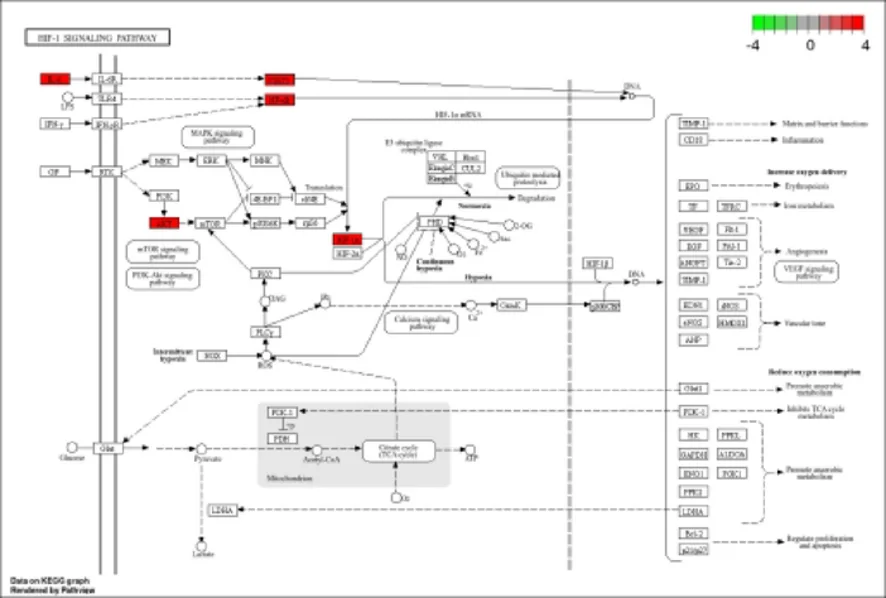
Identification of core signaling pathway against AKI from KEGG database

We performed GO and KEGG enrichment analyses on the GMN-ED. BP terms were associated with the regulation of interleukin-6, vascular endothelial growth factor production, and oxygen levels (Fig. 6A). CC terms were primarily enriched in the cytosol, interleukin-6 receptor complex, and NF-κB complex (Fig. 6B). MF terms were closely associated with interleukin-1 activity, cytokine binding activity, and NF-κB binding (Fig. 6C). KEGG enrichment analysis highlighted that the HIF-1 signaling pathway was most strongly implicated in the pathogenesis of AKI (Fig. 6D a). Targets in the GMN-ED were relevant to conditions including bone inflammatory diseases, ischemia, and immune disorders (Fig. 6E). These targets were also distributed across multiple cell types, such as macrophages, mononuclear cells, and epithelial cells (Fig. 6E). Figure 7 reflected the map of HIF-1. Taken together, our analysis of the GMN-ED provided specific insights into the involvement of inflammatory cytokines and the HIF-1 signaling pathway in the development of AKI.

### The MCODE based cluster analysis of GMN-ED

**Fig. 8:**
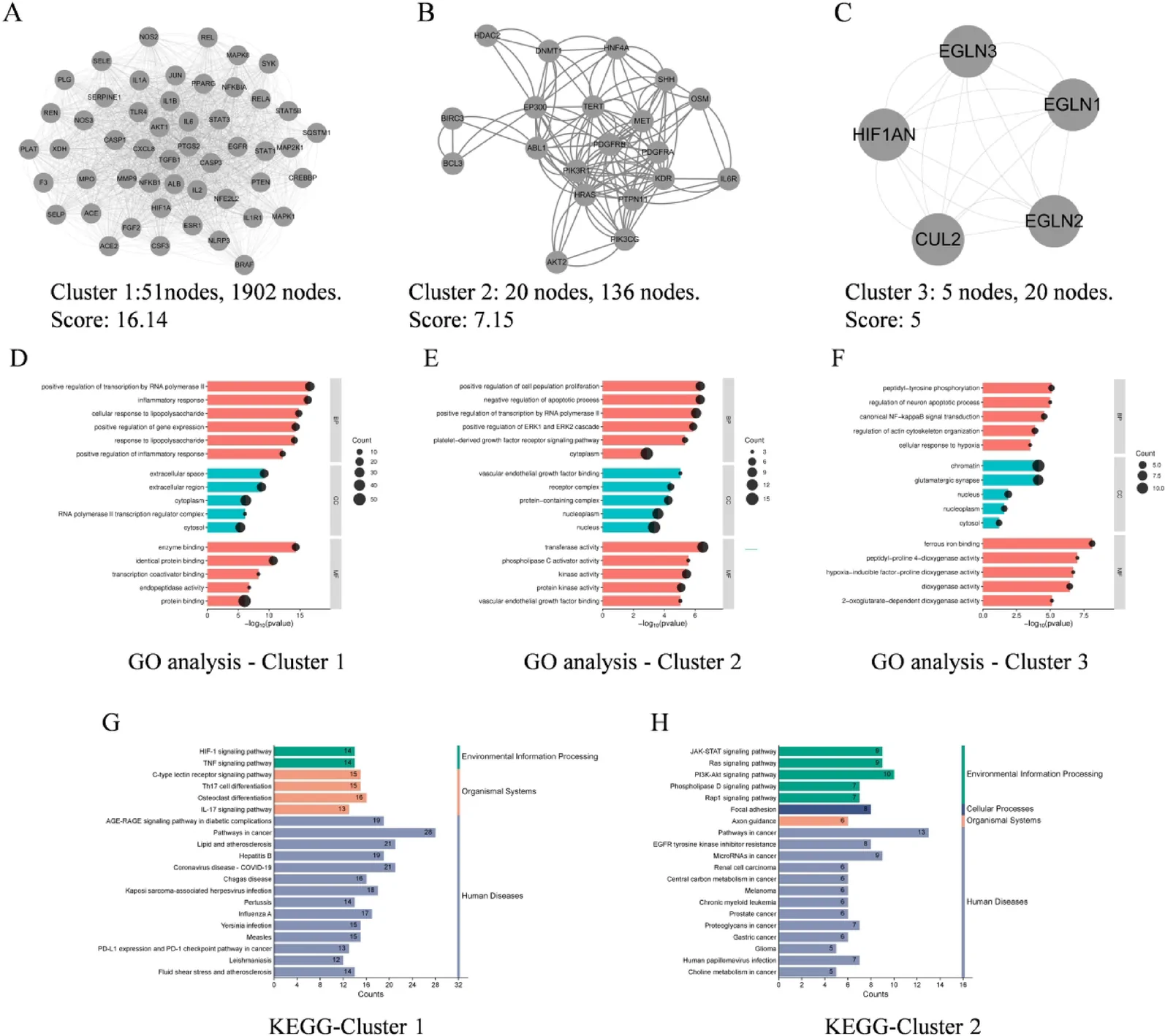
The cluster analysis of GMN. **A**–**C** The top 3 clusters based on MCODE analysis. **D**–**F** The GO analysis of the top 3 clusters respectively. **G** The KEGG enrichment analysis of Cluster 1. **H** The KEGG enrichment analysis of Cluster 2

To further investigate the therapeutic module of GMN-ED, cluster analysis was performed by using the MCODE algorithm. The result indicated that GMN-ED could be divided into 3 distinct cluster (Fig. 8A–C). Cluster 1 contained 51 nodes connected by 1902 edges with a score of 16.14. Cluster 2 consisted of 20 nodes and 136 edges with a score of 7.15. Cluster 3 was composed of 5 nodes and 20 edges with a score of 5. The GO enrichment analysis of these clusters offered us a specific insight into their functional roles (Fig. 8D–F). The GO items of the cluster 1 were mostly enriched in the signal transduction, inflammation, cytosol, cytoplasm, RNA polymerase and enzyme binding. The GO items of the cluster 2 were mostly associated with the regulation of cell proliferation, apoptotic process, ER1 and ER2 cascade, nucleus, kinase activity and vascular epithelial growth factor binding. The GO items of the cluster 3 were mostly involved in the NF-kappaB signal transduction, cellular response to hypoxia, chromatin and hypoxia inducible factor proline dioxygenase activity. The pathway enrichment analysis revealed that cluster 1 and 2 involved in the regulation of HIF-1 pathway, TNF pathway, and JAK STAT pathway. These results confirmed that inflammation and HIF-1 pathway were considered the key regulatory mechanism of GMN-ED in the development of AKI.

### G-M-T-P network analysis and Molecular docking

**Fig. 9:**
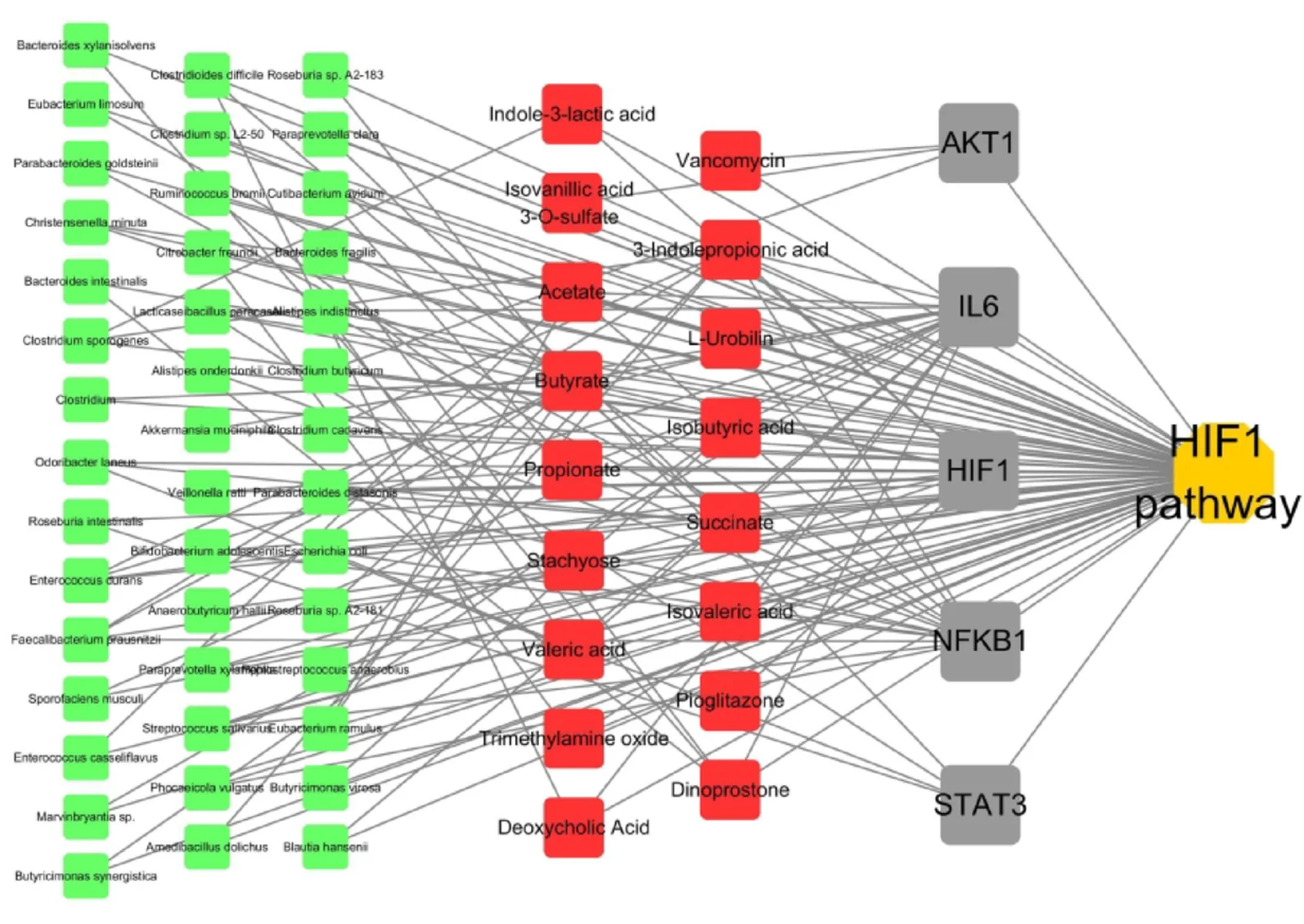
The Gut-Metabolites-Targets-Pathway network. The network revealing the mutual relationships among gut microbiota (green color), metabolites (red color), core targets (gray color) and key pathway (yellow color)

To further elucidate the interactions among the targets, metabolites, gut and pathway, we successfully constructed a “Gut Microbiota-Metabolites-Targets-Pathway” (G-M-T-P) network (Fig. 9). The green node represented GM, the red node represented metabolites, the gray node represented target, and the green node represented HIF-1 pathway. The G-M-T-P network consisted of 5 targets, 17 metabolites, 43 gut microbiota and 1 signaling pathway. Among these, IL6, NFKB1, and HIF-1 were most strongly involved in interactions with GM, metabolites and the HIF-1 signaling pathway, suggesting that these targets may exert therapeutic effects against AKI. We used the Network Analyzer tool to identify core metabolites. Degree scores indicated that acetate, butyrate, propionate, and 3-indolepropionic acid exhibited the highest values, identifying them as the top four metabolites.

**Fig. 10:**
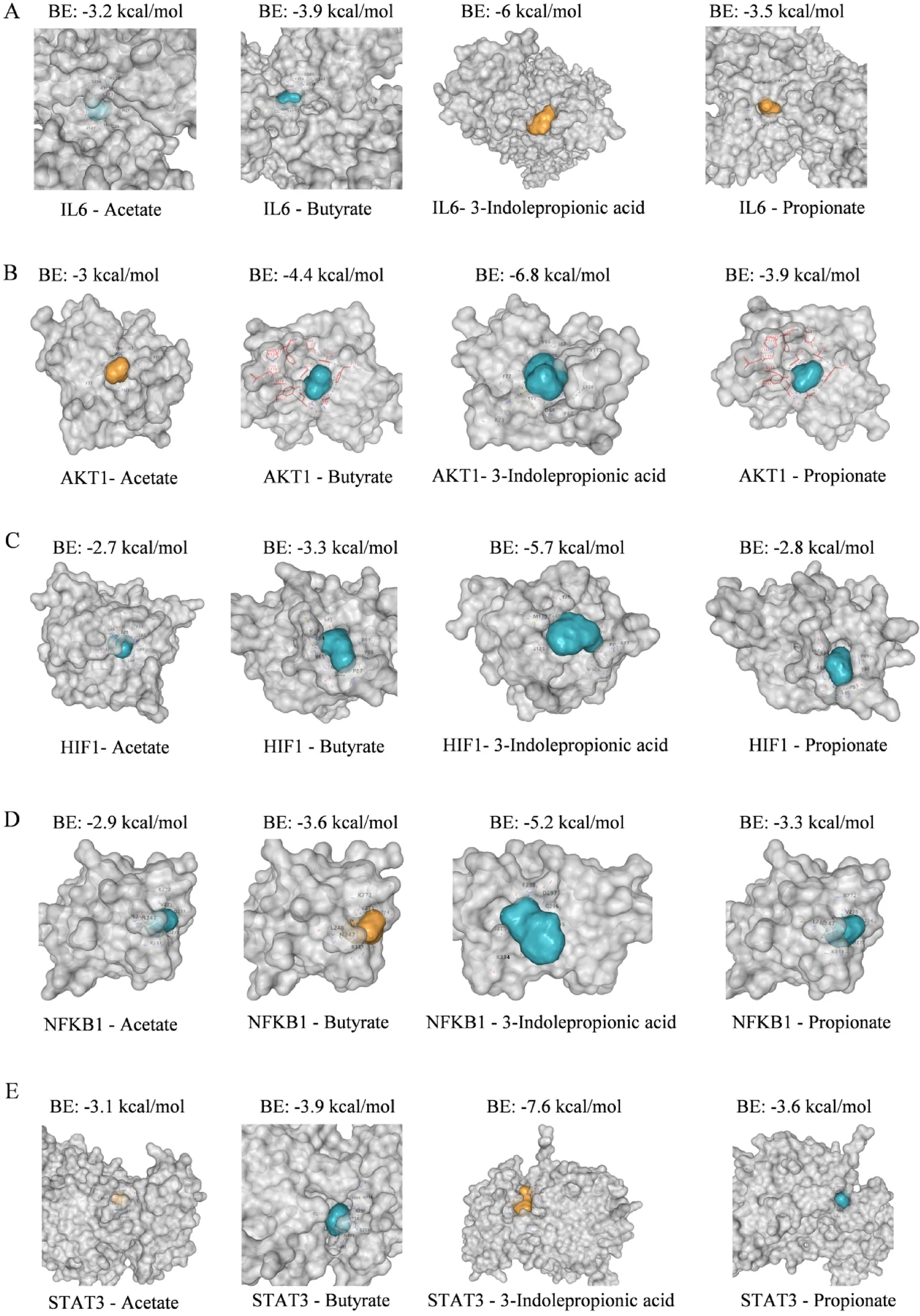
The visualization of molecular docking of core metabolites with core targets. **A** Molecular docking of Acetate, Butylate, 3-Indolepropionic acid and Propionate with IL6. **B** Molecular docking of Acetate, Butylate, 3-Indolepropionic acid and Propionate with AKT1. **C** Molecular docking of Acetate, Butylate, 3-Indolepropionic acid and Propionate with HIF1A. **D** Molecular docking of Acetate, Butylate, 3-Indolepropionic acid and Propionate with NFKB1. **E** Molecular docking of Acetate, Butylate, 3-Indolepropionic acid and Propionate with STAT3

Molecular docking could simulate the binding mode between active compounds and target proteins at the atomic level. By comparing the binding affinities between different active compounds and target proteins, molecular docking is capable of screening the core targets and compounds with the strongest binding affinity and the highest likelihood of exerting therapeutic effects, thereby enhancing the reliability of the research findings. Generally speaking, the binding energy < 0 kcal mol^− 1^ indicates that the complex of compound and target could exert biological function. As shown in in Fig. 10A–E, the results showed that the binding energy between metabolites and targets was less than 0 kcal mol^− 1^ indicating that they could bind tightly together. Of note, 3-indolepropionic acid displayed the highest binding affinity for the core targets, with binding energies below − 5 kcal mol^− 1^. Taken together, the metabolites could be considered as a potential candidate agent for AKI management by interacting with these targets to exert therapeutic effects.

## Discussion

Acute kidney injury (AKI) is a severe global health challenge characterized by a sudden decline in renal function (Rossiter et al. 2024; Pérez-Aizpurua et al. 2024). Multiple pathological processes, including autophagy, endoplasmic reticulum stress, oxidative stress, and inflammatory responses, are involved in the progression of AKI (Legrand et al. 2024). Although early diagnosis of AKI is feasible, current treatments still fail to effectively restore impaired renal function, imposing a substantial burden on healthcare systems and patients’ physical health. Therefore, achieving breakthroughs in therapeutic strategies for AKI holds profound clinical significance. In recent years, the protective effects of GM metabolites in AKI have been extensively studied (Jo 2021). However, their underlying protective mechanisms and corresponding regulatory effects remain largely unclear and require in-depth exploration. In the present study, we employed network pharmacology to decipher the therapeutic targets and molecular mechanisms of GM metabolites in the treatment of AKI.

The PPI network analysis successfully identified STAT3, HIF1A, NFKB1, IL6 and AKT1 as the core targets through which GM metabolites exerted beneficial effects on AKI. STAT3 is a critical transcription factor in the JAK-STAT signaling pathway, which plays a pivotal role in regulating the inflammatory response and is closely implicated in the pathogenesis of AKI (Ji et al. 2025). Lee et al. reported that STAT3 expression was upregulated in LPS-induced AKI, accompanied by the activation of pro-inflammatory macrophages and renal fibrosis (Lee et al. 2024). Treatment with a STAT3 inhibitor effectively alleviated inflammation and renal fibrosis. Accumulating evidence has demonstrated that the NF-кB is a key inflammatory regulator involved in pathogenesis of AKI through the modulation of immune response, apoptosis and cell survival (Huang et al. 2025). Chen et al. confirmed the deletion of NF-кB could attenuate renal tubular injury in AKI model by blocking the recruitment and infiltration of macrophages or inflammatory cytokines like IL1 and IL6 (Huang et al. 2025). AKT (protein kinase B) plays a central role in modulating multiple biological functions including cell survival, proliferation, stress responses, and inflammation (Yu et al. 2022; Guo et al. 2024). These functions are crucial to maintain the homeostasis within the renal microenvironment. During the development of AKI, the activation of AKT attenuated renal tubular apoptosis, reduced oxidative stress and inhibited excessive inflammatory responses (Liu et al. 2025). Conversely, the inhibition of AKT signaling aggravated renal injury and accelerated AKI progression. Taken together, targeted inhibition of STAT3, NF-кB and activation of AKT represents a promising therapeutic approach for the prevention and treatment of AKI.

The KEGG enrichment analysis highlighted that the HIF1 pathway was most likely involved in the pathogenesis of AKI. HIF1 pathway plays a critical role in the status of hypoxia (Wang et al. 2021; Ding et al. 2025). The kidney is highly sensitive to hypoxia. Hypoxia can induce renal injury through the activation of HIF1 pathway accompanied by inflammatory responses and macrophage polarization (Korbecki et al. 2021; Gao et al. 2023). Under moderate hypoxia condition, the activation of HIF1 pathway could exerts renoprotective effects by upregulating the expression level of VEGF. The protective mechanism was associated with the role of VEGF in promoting angiogenesis and maintaining the stability of renal microcirculation (Wang et al. 2022). In contrast, under severe or persistent hypoxia condition, the hyperactivation of HIF1 pathway leads to the activation of inflammatory related signaling pathways stimulating the release of pro-inflammatory factors such as IL-6 and TNF-α, and aggravating inflammatory infiltration and tubular damage in renal tissue (Spencer et al. 2021; Enescu et al. 2022; Natale et al. 2022). Clinical data demonstrated that plasma HIF-1 levels were significantly higher in pediatric patients with AKI than in those without AKI. In addition, the elevated expression of HIF-1 resulted in the upregulation of renal injury markers (serum creatinine, NGAL) and the decline in renal function (eGFR), confirming that the hyperactivation of HIF1 pathway serves as a crucial driver of AKI progression (Xu et al. 2025). Other pathways like VEGF and TNF pathway were also vital contributors to the pathogenesis of AKI (Abdelmageed et al. 2023). Renal microvascular endothelial injury represents a core pathological alteration in AKI. The hyperactivation of VEGF and TNF pathway could induce endothelial activation, increase vascular permeability, promote the release of pro-inflammatory cytokines and macrophage recruitment (Aanhold et al. 2022). These unfavorable consequences led to inflammatory responses and fibrogenesis in kidney, acting as a critical driver in the transition from AKI to CKD. Pharmacological inhibition of the HIF-1, VEGF, and TNF-α pathways could ameliorate hypoxia, suppress inflammation infiltration and fibrosis, which represented critical regulatory mechanisms to alleviate AKI.

The G-M-T-P network identified the top four metabolites, including acetate, butyrate, propionate and 3-indolepropionic acid, as the potential therapeutic metabolites against AKI. The acetate, butyrate, propionate are beneficial GM metabolites derived from SCFAs (Deleu et al. 2021; Sun et al. 2026). Recent studies emphasized the role of SCFAs in AKI treatment. The treatment with butyrate could improve renal function, reduce renal tubular injury and inflammation in the AKI model (Favero et al. 2024). Mechanistic investigations implied that the protective effect of butyrate was associated with inhibition of ERK1/2 and NF-κB pro-inflammatory signaling pathways and downregulation of histone deacetylation (HDAC) levels (Diep et al. 2025; Liu et al. 2026). The interplay between ROS (reactive oxygen species) and inflammation lead to renal injury. Acetate serving as a HDAC inhibitor could ameliorate renal cellular stress responses through epigenetic regulation (Kawabata et al. 2023). The excessive production of ROS caused by mitochondrial dysfunction is a key pathological features in cisplatin-induced kidney injury (Su et al. 2023). Experimental results indicated that treatment with acetate could markedly reduce ROS levels in renal tubular epithelial cells and upregulates the expression levels of antioxidant enzymes, including catalase (CAT) and superoxide dismutase 2 (SOD2). By enhancing cellular antioxidant capacity, acetate could block the ROS-mediated cascade of renal injury (Corte-Iglesias et al. 2024). 3-indolepropionic acid is another beneficial GM metabolite. The serum levels of 3-indolepropionic acid were markedly decreased in patients with diabetic kidney disease (DKD) and DKD model mice compared with the normal control group, suggesting that 3-indolepropionic acid may serve as a potential biomarker for the diagnosis of DKD (Zeng et al. 2025). The animal experiments revealed that 3-indolepropionic acid intervention could significantly reduce urinary albumin levels in DKD mice, maintain the integrity of the glomerular filtration barrier and alleviate renal fibrosis as well as glomerular injuries. The protective mechanism of 3-indolepropionic acid is associated with maintaining the normal function of mitochondria and ROS reduction (Zeng et al. 2025). The renoprotective effects of propionate on suppressing inflammation and fibrosis were mainly mediated by the inhibition of HDAC and activation of G protein-coupled receptors (GPR41/43) (Yao et al. 2026). In a folic acid-induced AKI animal model, propionate treatment could reduce the expression of renal injury markers (KIM-1 and NGAL) and the production of collagen (Corte-Iglesias et al. 2024; Wang et al. 2024). Meanwhile, propionate treatment could mitigate inflammation by reducing monocyte infiltration into renal tissues.

This study integrating network pharmacology and bioinformatics proposes a novel regulatory mechanism of AKI from the perspective of how GM metabolites affecting the progression of AKI. However, it should be noted that there are still some limitations in this study. Firstly, although network pharmacology helped us identify the core GM metabolites and targets, their interactions have not yet been verified by experiments. Second, the results presented in this study were generally based on database collection without in vivo and in vitro experimental validations, which restrict its application to the clinical treatment. Third, while involvement of key signaling pathways were confirmed by KEGG enrichment analysis, yet their actual function has not been testified. The solutions to overcome these limitations is to conduct more experimental validations to ensure its accuracy and safety in future research.

## Conclusions

Our study suggests that the employment of network pharmacology could establish a powerful platform for elucidating the complex mechanism underlying the onset of disease. The results revealed that GM metabolites could exert protective effects on AKI. The core targets (STAT3, HIF1A, NFKB1, IL6 and AKT1) were involved in the regulation of HIF-1 signaling pathways, which modulated inflammatory and immune responses. Overall, the GM metabolites could alleviate AKI through the inhibition of inflammatory response through the interaction with multi targets and pathways, which could be used for candidate drug development in future AKI treatment.

## Data Availability

The data are available upon request.

## Abbreviation

PPI: Protein Protein interaction network
GO: Gene ontology
KEGG: Kyoto encyclopedia of genes and genomes
SMILES: Simplified molecular input line entry system
SCFAs: Short-chain fatty acids
AKI: Acute kidney injury
STAT3: Signal transducer and activator of transcription 3
HIF1A: Hypoxia‑inducible factor‑1A
AKT1: AKT serine/threonine kinase 1
IL6: Interleukin 6
NFKB1: Nuclear factor kappa‑B subunit 1
